# Socioeconomic inequalities in treatment and relative survival among patients with diffuse large B-cell lymphoma: a Hong Kong population-based study

**DOI:** 10.1038/s41598-021-97455-5

**Published:** 2021-09-09

**Authors:** Shing Fung Lee, Andrew M. Evens, Andrea K. Ng, Miguel-Angel Luque-Fernandez

**Affiliations:** 1 Department of Clinical Oncology, The University of Hong Kong, Hong Kong, China; 2 Department of Clinical Oncology, The University of Hong Kong–Shenzhen Hospital, Shenzhen, China; 3 Department of Clinical Oncology, Tuen Mun Hospital, New Territories West Cluster, Hospital Authority, Hong Kong, China; 4 Rutgers Cancer Institute of New Jersey, New Brunswick, New Jersey USA; 5 Department of Radiation Oncology, Brigham and Women’s Hospital and Dana-Farber Cancer Institute, Harvard Medical School, Boston, MA USA; 6 Department of Non-Communicable Disease Epidemiology, Cancer Survival Group, London School of Hygiene and Tropical Medicine, London, UK; 7 Department of Non-Communicable Disease and Cancer Epidemiology, Instituto de Investigacion Biosanitaria de Granada (Ibs.GRANADA), University of Granada, Granada, Spain

**Keywords:** Haematological cancer, Health policy, Health services

## Abstract

The influence of socioeconomic status (SES) on access to standard chemotherapy and/or monoclonal antibody therapy, and associated secular trends, relative survival, and excess mortality, among diffuse large B-cell lymphoma (DLBCL) patients is not clear. We conducted a Hong Kong population-based cohort study and identified adult patients with histologically diagnosed DLBCL between 2000 and 2018. We examined the association of SES levels with the odds and the secular trends of receipt of chemotherapy and/or rituximab. Additionally, we estimated the long-term relative survival by SES utilizing Hong Kong life tables. Among 4017 patients with DLBCL, 2363 (58.8%) patients received both chemotherapy and rituximab and 740 (18.4%) patients received chemotherapy alone, while 1612 (40.1%) and 914 (22.8%) patients received no rituximab or chemotherapy, respectively. On multivariable analysis, low SES was associated with lesser use of chemotherapy (odd ratio [OR] 0.44; 95% CI 0.34–0.57) and rituximab (OR 0.41; 95% CI 0.32–0.52). The socioeconomic disparity for either treatment showed no secular trend of change. Additionally, patients with low SES showed increased excess mortality, with a hazard ratio of 2.34 (95% CI 1.67–3.28). Improving survival outcomes for patients with DLBCL requires provision of best available medical care and securing access to treatment regardless of patients’ SES.

## Introduction

Diffuse large B-cell lymphoma (DLBCL) is the most common type of non-Hodgkin lymphoma (NHL) globally, constituting 25–40% of all cases in different geographic regions^1–4^. The median survival time without active treatment is less than 1 year^5^. However, with effective modern therapeutic strategies, a 5-year survival exceeding 60–65% is achieved, according to US population-based data^1,6^.

Despite advancements in treatment, the role of socioeconomic status (SES) on access to effective treatment among DLBCL patients remains controversial. In a study using the National Cancer Database from the United States on patients with DLBCL, those with low SES were significantly less likely to have received chemotherapy or chemoimmunotherapy^7^. However, in another population-based study on patients with DLBCL from the Netherlands, which offers free access to health care, no disparities in treatment and survival were observed in patients with low SES^8^. Hong Kong has a healthcare system that offers both public and private medical care options, akin to that in countries such as United Kingdoms and Singapore^9,10^. It remains unclear whether optimal treatment is provided across socioeconomic groups.

An improved understanding of the socioeconomic disparities in access to treatment can provide insight into development of innovative strategies that extend effective therapies to more patients and improve survival. We aimed to determine the association between SES and access to therapy, its secular trend, as well as the long term DLBCL relative survival and excess mortality in a population-based study from Hong Kong.

## Materials and methods

### Population, settings and data

Data were retrieved from the Clinical Data Analysis and Reporting System (CDARS), which is a territory-wide electronic database operated by the Hospital Authority of Hong Kong. The Hospital Authority is the sole public healthcare service provider and covers approximately 90% of all medical care in Hong Kong, which has a population of around 7.5 million^11^. Data, including demographics, hospitalizations, diagnoses, treatment, and causes, times, and dates of death, are recorded in CDARS. The International Classification of Diseases, Ninth Revision was used for disease coding. Previous studies have demonstrated positive and negative predictive values exceeding 90%^12,13^. High-quality population-based studies have been conducted based on the data retrieved from CDARS^13–15^.

**Figure 1:**
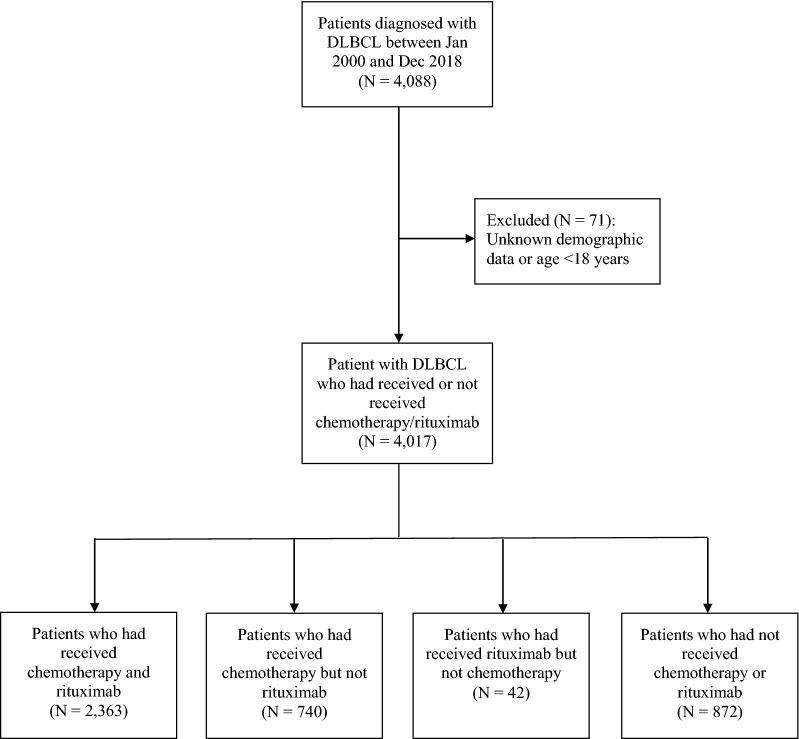
Flowchart outlining the inclusion and exclusion criteria, Hong Kong, 2000–2018 (N = 4017). The light grey bands represent the 95% confidence interval bands. DLBCL, diffuse large B-cell lymphoma.

We identified patients with DLBCL who were histologically diagnosed in both inpatient and outpatient settings between January 1, 2000 and December 31, 2018 from CDARS. The Research Ethics Committee, New Territories West Cluster, Hospital Authority, Hong Kong approved this study and waived patient consent requirement (reference no: NTWC/REC/19085). This research project was conducted in accordance with the 1964 Declaration of Helsinki and its later amendments. Figure 1 shows the case selection flowchart and the final number of patients who constituted the study population^16^. Patients were excluded if they had uncertain demographic data or younger than 18 years. The receipt of systemic treatment (therapies that potentially affect the entire body) was defined as either having received chemotherapy and/or rituximab. These systemic treatments might be used alone or in combination with radiotherapy.

### Variables included in the analysis

We included the following factors that potentially are associated with both the probability of receipt of systemic treatment and patient’s SES: DLBCL patient’s age at diagnosis, sex, comorbidity status, serum lactate dehydrogenase (LDH), and year of diagnosis (categorized into 4 similarly sized year ranges). We dichotomized age (> 60 years versus ≤ 60 years) and serum LDH (elevated vs normal, local cut-off value 250 units per liter) according to the DLBCL prognostic scoring system (i.e., the International Prognostic Index)^17^.

SES was defined based on the need for comprehensive social security assistance (CSSA). CSSA is a basic welfare scheme in Hong Kong that provides supplementary payments to households that cannot support themselves financially^18^. The scheme aims to bring the income of such individuals and families up to a prescribed level to meet their basic needs of living. The CSSA recipients are also subsidized in medical care. We extracted information regarding this medical financial assistance at household level as a surrogate for SES.

Comorbid conditions was measured using the Royal College of Surgeons (RCS) adaptation of the Charlson Comorbidity Index (Supplementary Method S1)^19,20^.

### Statistical analysis

Descriptive statistics for demographics and RCS-modified Charlson score, serum LDH, year of diagnosis were generated for the DLBCL patients. Continuous variables were presented as medians with interquartile ranges and compared using the rank-sum test, while categorical variables were presented as percentages and compared using the chi-squared test to describe the differences between SES groups. Factors associated with the initiation of systemic treatment (i.e., chemotherapy and/or rituximab) were evaluated by univariate and multivariate generalized linear regression models using a binomial family and logit link^21^. The multivariate models were adjusted for SES, age at lymphoma diagnosis (> 60 years vs ≤ 60 years), sex (male vs female), RCS Comorbidity score, serum LDH (elevated vs normal), and year of diagnosis. To explore the temporal trends in the odds of the initiation of systemic treatment by SES we developed a second multivariate model including all the above covariates plus the interaction between the SES and the year of diagnosis. The goodness of fit for the main models was assessed based on the analysis of the residuals and the deviance statistic^21^. Then, the marginal probabilities of having received systemic treatment over time by SES status were calculated from the predictions of the fitted models and averaged over the observed values of the covariates in the analyses. They are interpreted as the contrast in the mean predicted probability of systemic treatment for those DLBCL patients with low versus high SES over time^22,23^. Finally, we evaluated the long-term excess mortality and relative survival of DLBCL patients accounting for the competing risk of death for other causes by SES at 15 years (see Supplementary Method S2). We used Stata v.16.1 (StataCorp, College Station, Texas, U.S.) for statistical analyses^24^.

## Results

The characteristics of the analyzed DLBCL cohort (N = 4017) are detailed in Table 1. The median age at diagnosis was 65 years (interquartile range 54–76 years) and 55.9% were male. As of September 30, 2019, the median follow-up time from index date for the entire lymphoma cohort was 6.9 years (minimum 0.8 to maximum 15.0 years). Overall, 1848 patients died during the study period and we there were 18,836 person-years of follow-up. Among 4017 patients, 2363 (58.8%) received both chemotherapy and rituximab, and 740 (18.4%) received chemotherapy alone, while 1612 (40.1%) and 914 (22.8%) received no rituximab or chemotherapy, respectively. Collectively, patients who had high SES were younger (median age 64 years vs. 75 years for high vs. low SES, respectively, rank-sum test p < 0.001) and had less comorbidities (Chi-square test p < 0.001). Older patients generally had more comorbidities (Supplementary Table S1).

**Table 1:** Characteristics of diffuse large B-cell lymphoma patients, Hong Kong, 2000–2018 (N = 4017).

Characteristics	All lymphoma patients (N = 4017)	Lymphoma patients categorized by receipt of chemotherapy (N = 4017)		*p*	Lymphoma patients categorized by receipt of rituximab (N = 4017)		*p*
		Chemotherapy (N = 3103)	No chemotherapy (N = 914)		Rituximab (N = 2405)	No rituximab (N = 1612)	
Age at diagnosis, median (interquartile range), years	65 (54–76)	63 (53–73)	75 (63–82)	< 0.001	63 (54–73)	70 (56–79)	< 0.001
**Sex, No. (%)**				0.690			0.022
Male	2247 (55.9)	1741 (56.1)	506 (55.4)		1310 (54.5)	937 (58.1)	
Female	1770 (44.1)	1362 (43.9)	408 (44.6)		1095 (45.5)	675 (41.9)	
**SES, No. (%)**				< 0.001			< 0.001
Lower	396 (9.9)	241 (7.8)	155 (17.0)		171 (7.1)	225 (14.0)	
Higher	3621 (90.1)	2862 (92.2)	759 (83.0)		2234 (92.9)	1387 (86.0)	
**Race, No. (%)**				0.491			0.248
Chinese	3847 (95.8)	2968 (95.6)	879 (96.2)		2296 (95.5)	1551 (96.2)	
Non-Chinese	170 (4.2)	135 (4.4)	35 (3.8)		109 (4.5)	61 (3.8)	
**RCS Co-morbidity Scores, No. (%)**				< 0.001			< 0.001
0	1988 (49.5)	1587 (51.1)	401 (43.9)		1226 (51.0)	762 (47.3)	
1	1183 (29.4)	932 (30.0)	251 (27.5)		731 (30.4)	452 (28.0)	
≥ 2	846 (21.1)	584 (18.8)	262 (28.7)		448 (18.6)	398 (24.7)	
**Serum lactate dehydrogenase, No. (%)**				< 0.001			< 0.001
Normal	1553 (43.1)	1333 (45.4)	220 (32.9)		1138 (48.4)	415 (33.2)	
Elevated	2050 (56.9)	1601 (54.6)	449 (67.1)		1213 (51.6)	837 (66.8)	
**Year of diagnosis, No. (%)**				< 0.001			< 0.001
2000–2004	787 (19.6)	453 (14.6)	334 (36.5)		129 (5.4)	658 (40.8)	
2005–2009	995 (24.8)	800 (25.8)	195 (21.3)		587 (24.4)	408 (25.3)	
2010–2014	1220 (30.4)	1026 (33.1)	194 (21.3)		924 (38.4)	296 (18.4)	
2015–2018^a^	1015 (25.3)	824 (26.6)	191 (20.9)		765 (31.8)	250 (15.5)	

*RCS* Royal College of Surgeons, *SES* socioeconomic status.

^a^Four years only.

On univariate analysis (Table 1), older age at diagnosis, low SES, RCS co-morbidity scores ≥ 2, elevated LDH, and earlier calendar year of diagnosis were associated with lower likelihood of receipt of chemotherapy and/or rituximab.

**Figure 2:**
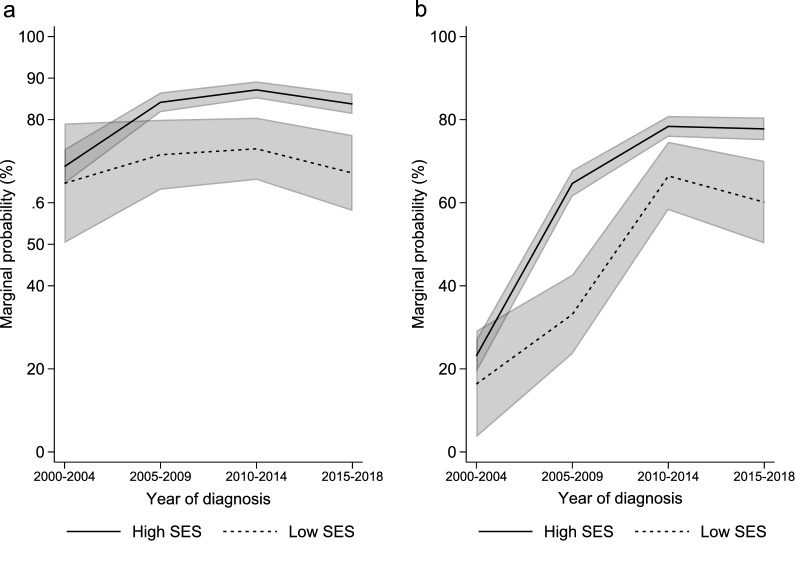
Secular trends of the probability of receiving (**a**) chemotherapy and (**b**) rituximab respectively among DLBCL patients by SES in Hong Kong, 2000–2018 (N = 4017). The light grey bands represent the 95% confidence interval bands. *SES* socioeconomic status.

In the multivariate regression models (Table 2), low SES was associated with a significantly lower odds of receiving any chemotherapy (OR 0.44; 95% CI 0.34–0.57; p < 0.001) or rituximab (OR 0.41; 95% CI 0.32–0.52; p < 0.001). Patients who were above 60 years of age, and had elevated LDH were also independently less likely to receive chemotherapy or rituximab. Additionally, patients with RCS comorbidity score ≥ 2 were less likely to receive chemotherapy (OR 0.80; 95% CI 0.64–0.99; p = 0.044), and male patients were less likely to receive rituximab (OR 0.8; 95% CI 0.68–0.93; p = 0.004). Patients diagnosed in recent years were more likely to receive either treatment. We found no evidence of temporal change in the socioeconomic gap on the odds of receiving chemotherapy and rituximab during the analyzed period (Supplementary Table S2). The marginal probability that a patient in low and high SES received chemotherapy were 64.7% and 68.7%, respectively in 2000–2004; the probabilities increased to 67.1% and 83.8%, respectively in 2015–2018 (Fig. 2a). For rituximab, the marginal probability for lower and higher SES were 16.4% and 23.1% respectively in 2000–2004, and the probabilities increased to 60.1% and 77.8% respectively in 2015–2018 (Fig. 2b). The adjusted odds ratios of rituximab use in 2015–2018 were 0.76 (95% CI 0.43–1.33; p = 0.334), 3.12 (95% CI 1.70–5.75; p < 0.001), and 8.04 (95% CI 2.85–22.70; p < 0.001), when compared with years 2010–2014, 2005–2009, and 2000–2004 respectively.

**Table 2:** Factors for initiation of any chemotherapy and rituximab by multivariable logistic regression models among patients with diffuse large B-cell lymphoma, Hong Kong, 2000–2018 (N = 4017).

Characteristics^a^	Multivariable logistic regression models			
	Use of any chemotherapy vs none		Use of rituximab vs none	
	OR (95% CI)	*p*	OR (95% CI)	*p*
SES (lower vs higher)	0.44 (0.34–0.57)	< 0.001	0.41 (0.32–0.52)	< 0.001
Age at diagnosis (> 60 vs ≤ 60)	0.41 (0.34–0.51)	< 0.001	0.72 (0.61–0.85)	< 0.001
Sex (male vs female)	0.99 (0.83–1.18)	0.916	0.80 (0.68–0.93)	0.004
RCS comorbidity score^b^ (One vs Zero)	1.11 (0.90–1.37)	0.345	1.10 (0.92–1.32)	0.297
(Two vs Zero)	0.80 (0.64–0.99)	0.044	0.84 (0.69–1.02)	0.087
Serum lactate dehydrogenase (elevated vs normal)	0.66 (0.55–0.79)	< 0.001	0.66 (0.57–0.78)	< 0.001
Year of diagnosis (2005–2009 vs 2000–2004)	2.39 (1.84–3.10)	< 0.001	5.92 (4.58–7.66)	< 0.001
(2010–2014 vs 2000–2004)	2.98 (2.30–3.87)	< 0.001	12.92 (9.93–16.79)	< 0.001
(2015–2018 vs 2000–2004)	2.26 (1.74–2.93)	< 0.001	12.03 (9.20–15.73)	< 0.001

*CI* confidence intervals, *OR* odds ratio, *RCS* Royal College of Surgeons, *SES* socioeconomic status.

^a^Adjusted for all the covariates included in Table 1 except race, because most of the patients were Hong Kong Chinese.

^b^Included myocardial infarction, congestive heart failure, peripheral vascular disease, cerebrovascular disease, diabetes mellitus, dementia, chronic pulmonary disease, rheumatic disease, liver disease, hemiplegia/paraplegia, renal disease, acquired immune deficiency syndrome/human immunodeficiency viral infection.

**Figure 3:**
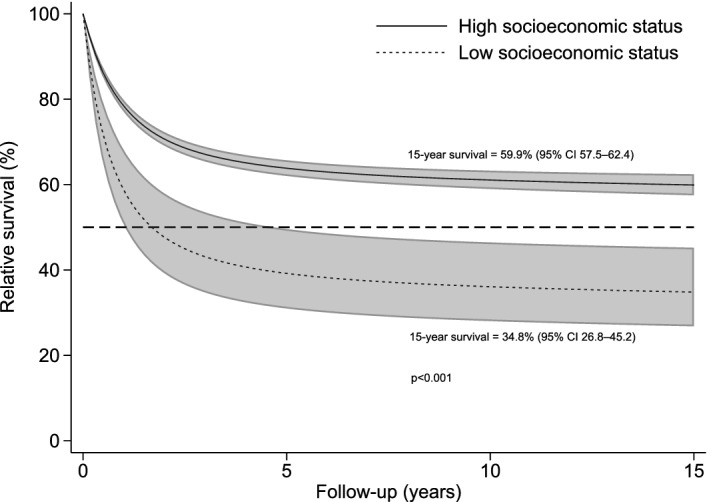
Relative **s**urvival curves of DLBCL by SES groups, produced under the framework of relative survival using Hong Kong life tables, Hong Kong, 2000–2018 (*N* = 4017). The light grey bands represent the 95% confidence interval bands. *CI* confidence interval, *DLBCL* diffuse large B-cell lymphoma, *SES* socioeconomic status.

There was also strong evidence of social inequalities in relative survival by SES (Fig. 3). Compared with DLBCL patients with high SES (as baseline group), those with low SES showed an increased excess mortality (EM) risk at 15 years after cancer diagnosis (hazard ratio 2.34, 95% CI 1.67–3.28, p < 0.001). The 15-year probability of relative survival for low and high SES was 34.8% (95% CI 26.8–45.2%) and 59.9% (95% CI 57.5–62.4%) respectively. Supplementary Figure S1 and Table S3 show the cumulative incidence of mortality under the overall survival and the relative survival framework respectively. We detected an increasing survival gap between patients with high and low SES over time.

## Discussion

In this population-based study, we found evidence of disparity related to SES for access to cancer treatment amongst DLBCL patients in Hong-Kong. After controlling for age, number of co-morbidities and other factors, SES remained significantly associated with the odds of receiving systemic therapy. Patients with a low SES had a 56% lower odds of receiving chemotherapy, and a 59% lower odds of receiving rituximab, compared with patients with a higher SES.

The existence of SES disparities in the use of cancer treatment and in mortality among NHL patients have been reported in many US studies^7,25–27^. In countries with a universal or near-complete health care coverage system, discrepancy in SES in access to treatment and survival in NHL patients are more controversial. A UK population-based study using area-based socioeconomic status revealed no significant socioeconomic variations across treatment access and survival outcomes^28^. However, in a Danish population-based study socioeconomic inequality in survival time among patients with NHL were detected by using individual-level markers of SES, despite no association between education or income and receipt of chemotherapy or immunotherapy was found^29,30^. The results from our study are distinct that we have found a SES disparity in receiving chemotherapy and rituximab in an Asian city where patients have both public and private care options. Furthermore, SES disparities in the access to treatment are associated with differences in long-term DLBCL survival estimates. The discrepancy in treatment might be explained by the inadequate insurance coverage and inability to cover contributing costs in care despite having subsidized health care in Hong Kong, resulting in inequalities in health care access^25,27^. We classified households who have financial hardship as having a low SES, while other studies variably assessed individual SES such as personal income and education level^29,30^, and ecological SES measures such as census-tract of residential area and neighborhood income and education level^7,8,25,31,32^. A possible reason for the variation in results among studies could be related to different definitions of SES among studies. Individual SES may relate to health behaviors including recognition of symptoms and adherence to treatment^33^, while aggregated measures of SES may be associated with availability of social and emotional support from peers or relatives, and ease of access to healthcare^34,35^. One should be careful to generalize our findings to their populations, and have to consider the peculiar organizational and geographic aspects of the structure of their health care system.

In our study, patients with significant comorbidity (RCS ≥ 2) were more commonly found in low SES group. These are consistent with findings from epidemiological studies^8,36^. We found that RCS ≥ 2 was significantly associated with a lower odd of receiving chemotherapy, but not rituximab. The presence of comorbidity that may interfere with intensive treatment and possible inequalities in treatment. Other hypotheses for patients with low SES having a lower chance of receiving treatment and shorter survival time include more advanced disease upon diagnosis^29^, inadequate long-term follow-up for these patients, and patient factors such as poorer nutritional status which might adversely affect treatment tolerance and subsequent survival. Further studies are necessary to assess the interaction between comorbidity and other patient-level variables such as race, nutrition, social support, emotional support, and informational support from family and health care providers on DLBCL treatment selection, in the context of the local healthcare system. It is notable, however, that the association between low SES and lower odds of receiving systemic therapy remains significant after controlling for RCS. We also found male patients to be significantly less likely to receive rituximab. A possible explanation could be a higher prevalence of hepatitis B infection among men^37,38^, affecting their eligibility for rituximab due to physician’s concern of the risk of hepatitis B reactivation^39^. The provision of prophylactic hepatitis B anti-viral agent was not mandatory and was based on self-pay system in public hospital until the recent 10 years or so^40^. However, we lack the data on the prevalence of hepatitis B and use antiviral prophylaxis in our cohort for exploring the relationship between the use of rituximab and anti-viral protection. The use of rituximab in our cohort has increased with time and stabilized from 2010 to 2014 onward, this could be related to start of subsidy program for the drug by government since late 2008^41^. Age beyond 60 years was inversely associated with receipt of treatment, this corroborated with previous findings^42–48^. Older patients generally have more comorbidities compared to younger patients (Supplementary Table S3) and poorer performance status^48,49^. Oncologists may steer away from recommended treatment for older patients due to a perception of decreased treatment tolerance and increased side effects, or a preference for avoiding toxicity to preserve quality of life^50,51^.

Our study has several limitations. The registry database is such that there is a lack of detailed information on lymphoma and its treatment such as stage at diagnosis, performance status, and the number of cycles of systemic treatments. Similar to other electronic medical record database studies, we also do not have data on such lifestyle factors as physical activity level and diet, and we were unable to quantify the effects of social support from peer and family on the treatment selections^52^. However, where possible we adjusted for components of the International Prognostic Index for DLBCL (age and serum LDH) as covariates in the regression models. We did not have information of SES in life tables, it is possible that we have underestimated the relative survival. Despite limitations, our study has several strengths. First, we analyzed a reasonably large and homogeneous Asian cohort over an almost 20-year time period. This allowed us to examine and account for the time trend of diffusion of treatment. Second, many population-based studies suffered from the effects of potential misclassification and under-reporting previously described in cancer registry studies^53,54^. Ascertainment of patients for this study was based on a diagnosis of DLBCL being rendered when the patient received care in outpatient or inpatient settings. This would reduce the possibility of selection bias because patients who are older, with more aggressive disease are more frequently diagnosed in inpatient setting^55^.

In this Asian population-based study involving adult DLBCL patients, we found that patients with low SES were less likely to receive chemotherapy and rituximab, in a region where the healthcare system is a mix of public and private medical options. Furthermore, low SES among DLBCL patients was associated with an increased excess mortality and reduced relative survival compared to those DLBCL patients with higher SES. Socioeconomic disparities on access to DLBCL treatment may contribute to the inferior survival estimates among patients with low SES. Improving cancer outcomes for patients with lymphoma requires provision of best available medical care and securing access to treatment regardless of patients’ SES. Further studies are needed to evaluate the barriers to cancer treatment among socioeconomically disadvantaged DLBCL patients in Hong-Kong.

## Supplementary Information

Supplementary Information.

## Data Availability

The datasets generated during and/or analyzed during the current study are available from the corresponding author on reasonable request.
