# Supplementary material for: Socioeconomic inequalities in treatment and relative survival among patients with diffuse large B-cell lymphoma: a Hong Kong population-based study
Source: Sci Rep. 2021 Sep 9;11:17950. doi: 10.1038/s41598-021-97455-5 (PMC8429768; doi:10.1038/s41598-021-97455-5)

Title

Socioeconomic Inequalities in Treatment and Relative Survival Among Patients with Diffuse Large B-Cell Lymphoma: A Hong Kong Population-Based Study

Authors' names

Shing Fung Lee, Andrew M. Evens, Andrea K. Ng, Miguel-Angel Luque-Fernandez

Supplementary Method S1. Royal College of Surgeons (RCS) adaptation of the Charlson Comorbidity Index

The RCS-modified Charlson score reduces the number of comorbidities to 14 by omitting a disease (peptic ulcer disease) and grouping categories (combining mild and severe liver disease, and diabetes with and without complications). The comorbidities (myocardial infarction, congestive heart failure, peripheral vascular disease, cerebrovascular disease, dementia, chronic pulmonary disease, rheumatic disease, liver disease, hemiplegia/paraplegia, renal disease, acquired immune deficiency syndrome/human immunodeficiency viral infection) were counted for each patient and categorized as no comorbidities, one comorbidity and two or more comorbidities.[1] The score does not assign weights to each comorbidity.[1]

References

1 Armitage, J. N. & van der Meulen, J. H. Identifying co-morbidity in surgical patients using administrative data with the Royal College of Surgeons Charlson Score. Br J Surg 97, 772-781, doi:10.1002/bjs.6930 (2010).

Supplementary Method S2. Long-Term Excess Mortality and Relative Survival of Diffuse Large B-Cell Lymphoma Patients.

We used a flexible parametric model including the restricted cubic splines of time with two knots and three degree of freedom.[1] The mortality information from Hong Kong life tables stratified by sex and age was included as an offset in the flexible parametric modelling in addition to all the other covariates described above.[2-4] The survival time was measured from the date of diagnosis for DLBCL patients and patients were followed until death or administrative censoring at 15 years after DLBCL diagnosis.[5] This approach is typically used when making long-term extrapolations as the patterns of cancer mortality and the other cause mortality are likely to be very different over time. The all-cause mortality rate can be broken into two constituent parts:

$$h(t) = hi*(t)+\lambda i(t) \quad (1)$$

where $hi(t)$ is the all-cause mortality, $hi*(t)$ is the background mortality typically obtained from population mortality rates stratified by age, sex and calendar year (and other general determinants of population mortality rates) and $\lambda i(t)$ is the excess mortality rate (computed as the ratio of hazards from the flexible parametric models, i.e., hazard ratios). The above equation can be transferred to the survival scale and rearranged to give the following relation:

$$Ri(t) = (\mathrm{Si(t)})/(\mathrm{Si*(t)}\ ) \ (2)$$

It means that the relative survival is the ratio of the all-cause survival and the expected survival in the background population.

References

1. Royston, P. & Lambert, P. C. Flexible Parametric Survival Analysis Using Stata: Beyond the Cox Model. (Stata Press, 2011).

2. Census and Statistics Department. Hong Kong Life Tables, 1971-2019, <https://www.censtatd.gov.hk/hkstat/sub/sp190.jsp?productCode=D5320184> (2020).

3. Pokhrel, A. & Hakulinen, T. How to interpret the relative survival ratios of cancer patients. European Journal of Cancer 44, 2661-2667, doi:10.1016/j.ejca.2008.08.016 (2008).

4. Lambert, P. C. & Royston, P. Further Development of Flexible Parametric Models for Survival Analysis. The Stata Journal 9, 265-290, doi:10.1177/1536867x0900900206 (2009).

5. NCI Dictionary of Cancer Terms: Relative Survival Rate, <https://www.cancer.gov/publications/dictionaries/cancer-terms/def/relative-survival-rate>

Supplementary Table S1. The Distribution of age and comorbidities Among Patients with Diffuse Large B-Cell Lymphoma, Hong Kong, 2000–2018 (N = 4,017)

	**Age at lymphoma diagnosis**	
**RCS Co-morbidity Scores, no. (%)**	**Age ≤ 60 years (N = 1,548)**	**Age > 60 years (N = 2,469)**
0	991 (64.0)	997 (40.4)
1	405 (26.2)	778 (31.5)
≥2	152 (9.8)	694 (28.1)

Abbreviation: RCS, Royal College of Surgeons

Chi-square test p <0.001

Supplementary Table S2. Multivariable Logistic Regression Models for Initiation of Any Chemotherapy and Rituximab with Interaction Between Socioeconomic Status and Year of Diagnosis Among Patients with Diffuse Large B-Cell Lymphoma, Hong Kong, 2000–2018 (N = 4,017)

	Multivariable logistic regression models			
	Use of any chemotherapy vs none		Use of rituximab vs none	
**Characteristics***	**OR (95% CI)**	***P***	**OR (95% CI)**	***P***
SES (lower vs higher)	0.82 (0.41 to 1.66)	0.590	0.64 (0.24 to 1.71)	0.379
Age at lymphoma diagnosis (>60 vs ≤60)	0.41 (0.34 to 0.51)	<0·001	0.72 (0.61 to 0.85)	<0.001
Sex (male vs female)	0.99 (0.83 to 1.78)	0.878	0.80 (0.68 to 0.93)	0.004
RCS Comorbidity score**				
(one vs zero)	1.11 (0.90 to 1.37)	0.344	1.10 (0.92 to 1.32)	0.288
(two vs zero)	0.79 (0.63 to 0.98)	0·034	0.83 (0.68 to 1.01)	0.068
Serum lactate dehydrogenase (elevated vs normal)	0.66 (0.55 to 0.80)	<0.001	0·66 (0·57 to 0·78)	<0·001
Year of diagnosis				
(2005–2009 vs 2000–2004)	2.51 (1.91 to 3.31)	<0.001	6.32 (4.84 to 8.25)	<0·001
(2010–2014 vs 2000–2004)	3.23 (2.44 to 4.27)	<0.001	12.69 (9.67 to 16.65)	<0·001
(2015–2018 vs 2000–2004)	2.44 (1.85 to 3.22)	<0.001	12.25 (9.28 to 16.17)	<0·001
Interactions between SES and year of diagnosis				
SES low x years 2005-2009	0.56 (0.24 to 1.29)	0.170	0.41 (0.14 to 1.20)	0.103
SES low x years 2010-2014	0.47 (0.20 to 1.06)	0.068	0.84 (0.29 to 2.40)	0.742
SES low x year 2015-2018	0.46 (0.20 to 1.07)	0.070	0.66 (0.22 to 1.92)	0.443

Abbreviations: CI, confidence intervals; OR, odds ratio; RCS, Royal College of Surgeons; SES, socioeconomic status

* Adjusted for all the covariates included in the table except race as most of the patients were Hong Kong Chinese.

[**] Included myocardial infarction, congestive heart failure, peripheral vascular disease, cerebrovascular disease, diabetes mellitus, dementia, chronic pulmonary disease, rheumatic disease, liver disease, hemiplegia/paraplegia, renal disease, acquired immune deficiency syndrome/human immunodeficiency viral infection.

Supplementary Table S3. The Cumulative Incidence of Mortality for Specific Time Points (1st, 2nd, 5th, 10th, and 15th year) Based on Overall Survival and Relative Survival Frameworks Respectively Among Patients with Diffuse Large B-Cell Lymphoma, Hong Kong, 2000–2018 (N = 4,017)

	**Cumulative Incidence of Mortality**	
	**Overall Survival Approach**	**Relative Survival Approach**
**High SES**	**% (95% CI)**	**% (95% CI)**
1st year	22.9 (21.6–24.3)	22.3 (21.0–23.7)
2nd year	31.2 (29.7–32.8)	29.9 (28.4–31.6)
5th year	41.3 (39.6–43.0)	36.2 (34.4–38.0)
10th year	49.1 (47.2–51.1)	38.9 (36.8–41.1)
15th year	53.7 (51.5–56.0)	40.1 (37.7–42.6)
**Low SES**		
1st year	44.0 (36.4–53.1)	42.4 (34.4–52.1)
2nd year	55.5 (47.4–64.9)	53.1 (44.6–63.2)
5th year	67.2 (59.4–75.9)	60.8 (52.2–70.8)
10th year	74.7 (67.6–82.6)	63.9 (55.4–73.7)
15th year	78.6 (71.9–85.8)	65.2 (56.7–75.0)

Abbreviation: SES, socioeconomic status

Supplementary Figure S1. Cumulative Incidence of Mortality of DLBCL by SES Groups under the Overall Survival and Relative Survival Frameworks, Hong Kong, 2000–2018 (N = 4,017). The color bands represent the 95% confidence interval bands. Abbreviations: CI, confidence interval. DLBCL, diffuse large B-cell lymphoma. SES, socioeconomic status

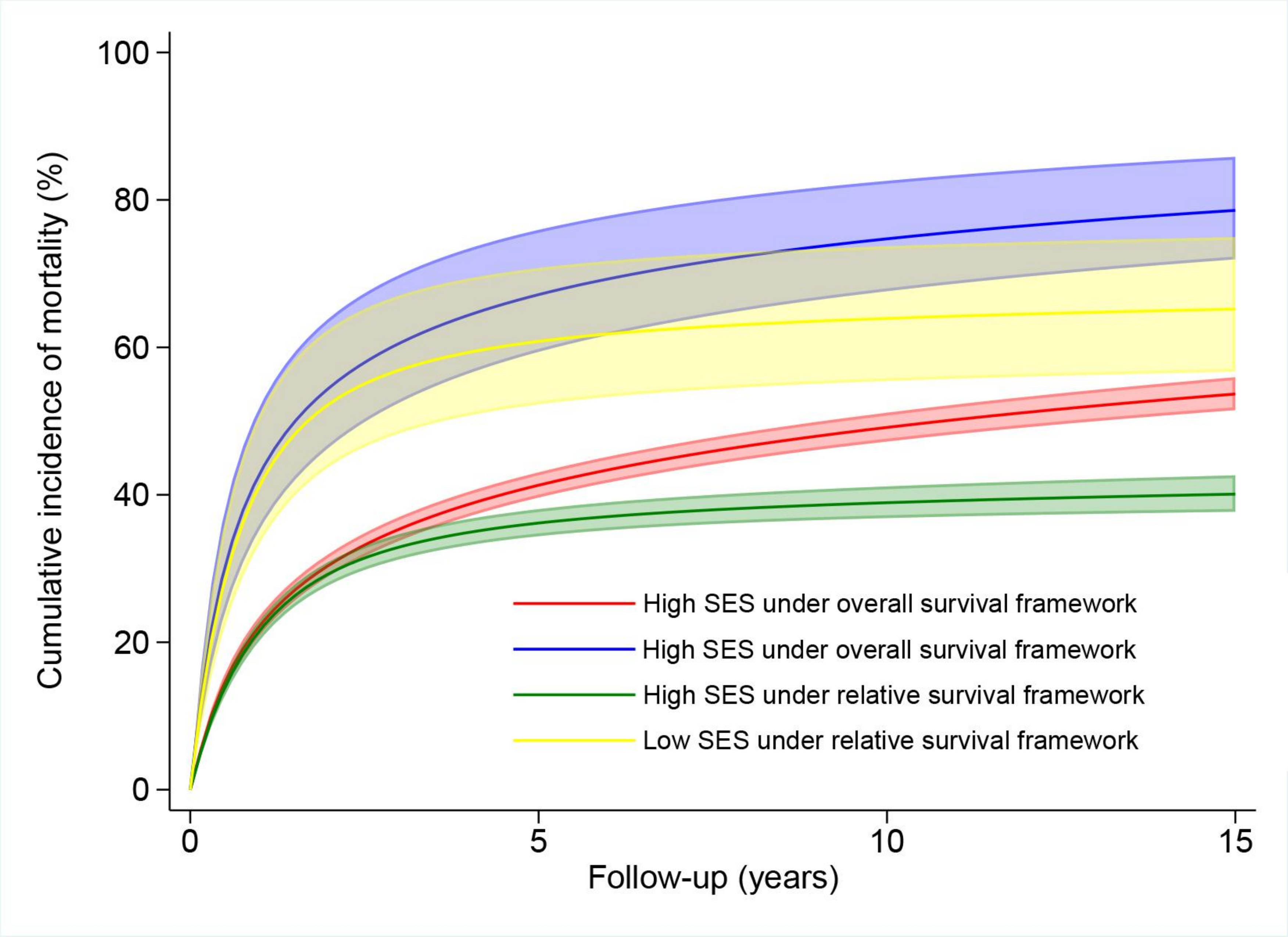

Supplement: Supplementary file 1 — Supplementary Information. [file 41598_2021_97455_MOESM1_ESM.pdf]
